# Beyond Classrooms: Community-Based Learning for Personal, Social, and Vocational Empowerment

**DOI:** 10.12688/f1000research.171496.3

**Published:** 2026-06-09

**Authors:** Ihwan Ridwan, Abdullah Sinring, Kartini Marzuki, Pangeran Paita Yunus, Zulkifli Zulkkifli, Yusriadi Yusriadi

**Affiliations:** 1 Universitas Negeri Makassar, Makassar, South Sulawesi, Indonesia; 2 Universitas Hasanuddin, Makassar, South Sulawesi, Indonesia

**Keywords:** Community-Based Learning, Empowerment, PRISMA, Personal Skills, Social Skills, Vocational Skills.

## Abstract

Community-Based Learning (CBL) situates learning with and within communities to build capabilities that extend beyond classrooms. This systematic review synthesizes evidence on the effectiveness of CBL for personal, social, and vocational empowerment. Following PRISMA 2020, we searched Scopus, Web of Science, ERIC, ProQuest, and Google Scholar (2010–2025) and conducted thematic synthesis complemented by bibliometric mapping. Of 8,395 records identified, 122 studies met inclusion criteria. Findings show consistent, positive effects on personal outcomes (e.g., self-confidence, agency, self-regulation) and social outcomes (e.g., teamwork, leadership, civic engagement), with vocational outcomes (e.g., employability, entrepreneurship, financial capability) also positive but more contingent on program design and market linkages. Effectiveness is strengthened by co-design with communities, cultural relevance, facilitator capability, cross-sector partnerships, and inclusive digital mediation; it is hindered by short funding cycles, heterogenous indicators, uneven governance, and equity/reporting gaps. The review further identifies three dominant conceptual and evaluative logics underpinning CBL implementation: capability-oriented, participation-based, and vocational transition approaches. The review integrates global evidence with local practices and SDG priorities (4, 8, 11) and proposes a unifying indicator logic to improve comparability across settings. We conclude that CBL functions as a systems intervention: when pedagogy is aligned with culture, governance, digital infrastructure, and labor-market connections, empowerment effects are stronger and more durable. Materials and extraction sheets are openly available on Zenodo to support transparency and reuse.

## Introduction

In the 21st century, education is increasingly recognized as extending beyond the boundaries of formal schooling. Global agendas such as the United Nations’ Sustainable Development Goals (SDGs) particularly Goal 4 (Quality Education), Goal 8 (Decent Work and Economic Growth), and Goal 11 (Sustainable Communities) underscore the need for inclusive and lifelong learning opportunities. Education is increasingly conceptualized as a lifelong process that integrates personal growth, social interaction, and cultural dimensions, fostering community engagement and offering solutions to context-specific challenges through structured educational initiatives ( Montes, 2025). Yet, despite progress in expanding access to formal education, traditional systems often remain limited by standardized curricula and credential-based outcomes, with insufficient capacity to address the diverse, context-specific needs of communities, especially in developing regions. Conventional education systems are frequently structured around uniform curricula that overlook the varied learning needs of students, especially those from economically disadvantaged or culturally diverse communities ( Beadle et al., 2022). This gap has stimulated growing interest in community-based learning (CBL) as an alternative and complementary approach that directly engages learners within their social, cultural, and economic environments.

CBL, often described as “learning with and within communities,” emphasizes participation, collaboration, communication, and contextual relevance. Community-based learning (CBL) engages students with community partners in addressing real-world challenges, within a collaborative setting that values diversity and fosters mutual respect ( Brown, 2024; Stephens, 2006). Unlike formal schooling, CBL draws upon local resources, networks, and cultural practices, positioning learning as a collective and situated process. Such approaches are particularly crucial for marginalized groups including rural farmers, informal laborers, and women’s cooperatives who may have limited access to formal education but require continuous skill development to thrive in rapidly changing societies. CBL combines practical learning with community needs, promoting collaboration between schools and local stakeholders, and has proven particularly effective in rural contexts where teachers mobilize networks to embed relevant skills ( Olayinka & Deniran, 2024). In these contexts, CBL not only facilitates knowledge transfer but also fosters empowerment, enabling individuals to exercise agency across personal, social, and vocational domains.

The empowerment outcomes of CBL can be broadly categorized into three skill domains. *Personal skills* encompass self-confidence, self-efficacy, responsibility, and discipline, which strengthen resilience and self-regulated learning. Students participating in CBL often demonstrate greater autonomy, confidence, and personal development gained through engagement with real-world contexts ( Ibrahim, 2010; Parker et al., 2009). *Social skills* include teamwork, cooperation, civic engagement, and leadership, fostering social cohesion and collective action Participation in CBL activities has been shown to enhance students’ interpersonal abilities and broaden their understanding of diverse communities ( Alsbury et al., 2020; Ibrahim, 2010). *Vocational skills* involve technical competencies, entrepreneurship, and financial literacy, directly contributing to employability and sustainable livelihoods. CBL links theory to practice through internships, projects, and service-learning ( Andrade et al., 2022). Scholars argue that these domains are interdependent for instance, personal confidence enhances collaboration, while social experiences of teamwork reinforce vocational capacities such as entrepreneurship. Despite this interdependence, much of the literature examines these domains in isolation, resulting in fragmented understandings and limited frameworks for capturing the holistic potential of CBL.

Several theoretical perspectives provide a foundation for interpreting these dynamics. Freire’s critical pedagogy highlights dialogical learning and education as a practice of freedom. Sen’s capability approach frames empowerment as the expansion of freedoms to achieve valued lives ( Hart, 2009), aligning with CBL’s emphasis on human flourishing. Wenger’s communities of practice conceptualize learning as participation in shared practices, where they facilitate informal learning and knowledge creation ( Wenger, 2009), while Knowles’ andragogy stresses relevance and self-direction for adult learners. Together, these perspectives demonstrate that CBL is more than a pedagogical method; it is a multidimensional process of empowerment that integrates individual agency, social identity, and vocational practice.

Over the past two decades, research on CBL has proliferated across education, vocational training, public health, and community development. In education, CBL has been applied to enhance literacy, numeracy, and digital skills. This method enables personalized learning paths, ensuring each student receives the necessary support to master essential skills ( Hokama & Kim, 2010; Wang et al., 2021). In vocational contexts, it has supported entrepreneurship and employability initiatives, while in social development it has strengthened civic participation and inclusion. Examples include community learning centers in Southeast Asia that reduce poverty through vocational training, East Java Community Learning Centers offer junior and senior high school programs, with success driven by manager factors and community involvement, enhancing education and reducing poverty ( Wiyono et al., 2021).

More recent studies further demonstrate CBL’s adaptability: Zamiri and Esmaeili (2024) identified peer mentoring and collaborative projects as effective strategies for personal and social development; Harmsel-Nieuwenhuis et al. (2025) showed how community-based life skills transfer into vocational capacities among vulnerable youth; and Kizys et al. (2025) highlighted how place-based CBL in rural STEM education improves both social engagement and career readiness. These findings reinforce the versatility of CBL while underscoring persistent fragmentation across domains.

Despite the expanding evidence on Community-Based Learning (CBL), several gaps remain that hinder a comprehensive understanding of its effectiveness. First, there has been little systematic synthesis of how CBL simultaneously contributes to personal, social, and vocational empowerment. Much of the literature highlights individual or community aspects in isolation without linking them to broader vocational outcomes ( Carr et al., 2015; Ciraso-Calí et al., 2023). Second, indicators of effectiveness are inconsistent ranging from participation rates to employment outcomes making cross-study comparisons difficult and limiting the ability to evaluate CBL’s overall impact ( Cao & Yu, 2023; Tommy et al., 2024). Third, contextual mediators such as cultural norms, governance systems, and digital infrastructures remain underexplored, even though evidence suggests they strongly shape community engagement and empowerment results ( Gemiharto & Yusup, 2023; Raharjo et al., 2021). Addressing these gaps is critical for advancing theoretical integration and improving the design of CBL initiatives.

Against this background, this review addresses an urgent need for a systematic synthesis of CBL’s effectiveness across multiple domains of empowerment. The study aims to (i) evaluate how CBL enhances personal, social, and vocational outcomes; (ii) identify indicators and instruments used to measure effectiveness; (iii) examine supporting and hindering factors; and (iv) map existing conceptual or evaluative models. By integrating evidence across fragmented strands, this review offers a comprehensive framework for assessing CBL’s empowerment potential.

The novelty of this study lies in two contributions. First, it advances an integrative perspective by analyzing personal, social, and vocational outcomes together, thereby addressing a longstanding gap in the literature. Second, it bridges global evidence and local practice, connecting international findings with context-specific initiatives such as Indonesia’s *Pusat Kegiatan Belajar Masyarakat (PKBM)* and indigenous community practices where learning is embedded in cultural life. This bridging enriches theoretical understanding while enhancing practical relevance for policymakers and practitioners. To our knowledge, this is the first systematic review to synthesize CBL outcomes across all three domains through a global local lens.

The remainder of this paper is structured as follows. Section 2 reviews the theoretical and conceptual foundations of CBL. Section 3 details the methodology following PRISMA 2020 guidelines. Section 4 presents the results of the synthesis. Section 5 discusses the findings in relation to theory, practice, and policy. Section 6 concludes with contributions, limitations, and recommendations for future research.

## Literature review

### Definition of Community-Based Learning (CBL)

Community-Based Learning (CBL) is defined as an educational approach that takes place *with* and *within* communities, grounded in participation, collaboration, communication, and contextual relevance ( Andrade et al., 2022; Brown, 2024). Unlike formal education, which often relies on standardized curricula, CBL leverages local resources, networks, and cultural practices to create inclusive, adaptive learning opportunities. Recent studies view CBL not merely as an *alternative* to schooling but as a complementary ecosystem that supports lifelong and situated learning ( Zamiri & Esmaeili, 2024).

### Personal, social, and vocational skills: Conceptual importance

Community-Based Learning (CBL) has consistently been associated with the development of personal skills, including confidence, self-efficacy, responsibility, and discipline. These competencies play a central role in fostering resilience and self-regulated learning, enabling students to manage their own learning processes, adapt to challenges, and ultimately achieve both academic and professional success ( Deed, 2010; Raath & Hay, 2016; Stapleton, 2025).

Equally important are the social skills cultivated through CBL, such as teamwork, cooperation, civic engagement, and leadership. These capacities contribute to building trust and strengthening social cohesion within communities. They also align with Wenger’s concept of communities of practice, which emphasizes that learning emerges through participation in collective practices and the development of shared identities ( Wenger, 2009).

In addition, CBL fosters vocational skills that are directly linked to employability and sustainable livelihoods. These include technical competencies, entrepreneurial abilities, and financial literacy, all of which prepare learners to respond effectively to industry demands. By emphasizing applied skills over abstract theoretical knowledge, CBL proves especially valuable in technical and vocational contexts, bridging the gap between students’ training and labor market expectations ( Boahin & Hofman, 2014; Zakaria et al., 2018).

Although personal, social, and vocational skills are interdependent where growth in one area often reinforces development in another research tends to examine them in isolation. For instance, vocational projects may neglect the cultivation of social skills, while civic initiatives rarely assess vocational outcomes. This fragmentation limits the development of integrative frameworks capable of capturing the holistic empowerment potential of CBL.

### Theoretical foundations

Community-Based Learning (CBL) is grounded in multiple theoretical traditions across education, sociology, and development. Freire’s critical pedagogy emphasizes education as a practice of liberation through dialogue and critical reflection ( Freire, 2000). Sen’s capability approach frames empowerment as the expansion of freedoms and opportunities to achieve valued life outcomes ( Sen, 1999). Wenger’s communities of practice highlights learning as social participation that builds identity and collective knowledge ( Wenger, 1998). Meanwhile, Knowles’ concept of andragogy underscores that adult learners require autonomy, relevance, and experiential application ( Knowles, 1980).

Table 1 summarizes the main theoretical perspectives that inform CBL, their implications for practice, and the gaps in their operationalization.

**Table 1.** Theoretical foundations of CBL.

Theory	Key principles	Implications for CBL	Gap in operationalization
**Freire Critical Pedagogy**	Education as liberation through dialogue	CBL fosters spaces for critical reflection and action	Rarely measured; empowerment often assumed, not assessed
**Sen Capability Approach**	Empowerment as expanded freedoms and opportunities	CBL enhances capabilities beyond skills acquisition	Few validated tools link CBL to capability outcomes
**Wenger Communities of Practice**	Learning as participation in shared practices	CBL builds identity and social capital via collective practice	Indicators of trust, leadership, and identity inconsistent
**Knowles Andragogy**	Adult learners need autonomy, relevance, and application	CBL aligns with adult-centered, experiential learning	Limited instruments to measure adult learner autonomy in CBL

### Previous findings across contexts

E Evidence from diverse regions illustrates the versatility of Community-Based Learning (CBL). In Asia, community learning centers (CLCs) have been central in promoting vocational training and improving rural livelihoods. For instance, Belete (2022) documents how CLCs provide literacy, technical training, and vocational skills that directly support community development, while Bun, Ueangchokchai, and Nopas (2025) show how Cambodian CLCs sustained vocational education during the COVID-19 crisis, thereby linking CBL to resilience in times of disruption.

In Africa, CBL has often been embedded in women’s cooperatives that foster solidarity and entrepreneurship. Raniga (2021) highlights how sewing cooperatives in South Africa significantly improved women’s income and social standing, while Arthur-Holmes (2024) conceptualizes collective action in informal cooperatives in the mining sector as a form of community empowerment. Complementing these findings, a UN Women (2023) initiative in Ethiopia demonstrated how revolving funds combined with basic business and life skills training transformed women’s livelihoods, underscoring the potential of CBL to address gendered dimensions of poverty.

In Western contexts, CBL has been applied to advance lifelong learning, civic participation, and sustainability goals. Lake (2021), for example, assessed the long-term impact of CBL on undergraduate students and found evidence of sustained development across civic, personal, and professional domains. These findings indicate that, beyond immediate skill gains, CBL can generate enduring outcomes aligned with broader social agendas in developed societies.

Recent systematic reviews reinforce these regional insights. Zamiri and Esmaeili (2024) identified digital platforms and collaborative projects as effective mechanisms for strengthening personal and social skills. Harmsel-Nieuwenhuis et al.(2025) demonstrated how life skills acquired in community contexts could transfer into vocational capacities among vulnerable youth. Similarly, Kizys et al. (2025) showed that place-based CBL in rural STEM education improved both career readiness and social engagement, emphasizing its adaptability to disciplinary and geographical variations.

Despite these advances, three persistent weaknesses limit the generalizability of findings. First, measurement inconsistency remains a challenge, as indicators vary widely from attendance rates to employment outcomes, hindering comparability across studies. Second, much of the research adopts a short-term focus, with limited longitudinal or quasi-experimental designs capable of capturing the durability of CBL’s impacts. Third, contextual mediators such as cultural norms, governance structures, and digital infrastructures are acknowledged but rarely examined systematically, despite their potential to shape program outcomes significantly.

### Identified gaps and multidisciplinary insights

Synthesizing the literature across disciplines reveals several enduring limitations that constrain a comprehensive understanding of Community-Based Learning (CBL). First, there is an integration gap, as few studies propose holistic frameworks that explicitly link personal, social, and vocational outcomes. Many studies concentrate on certain outcomes without incorporating them into a holistic paradigm ( Alsbury et al., 2020; Rojo, 2025). Second, a measurement gap persists, with an absence of validated, theory-driven indicators capable of capturing empowerment across domains. Validated, theory-driven measures that adequately capture empowerment are lacking. Current research underscores the necessity for defined metrics to accurately assess empowerment ( Lauzier et al., 2014; Meyerson et al., 2024). Third, a causality gap is evident, since most studies rely on cross-sectional designs, offering limited longitudinal evidence and weak causal inference. These designs, although common, provide insufficient longitudinal evidence and weak causal inference, hence limiting the capacity to derive robust findings regarding the long-term effects and causal linkages within CBL contexts ( Shahar & Shahar, 2013; Spector, 2019). Fourth, an equity gap emerges, given the insufficient disaggregation of findings by gender, age, disability, or socioeconomic status. To rectify the equity gap, it is essential to disaggregate data by diverse demographic characteristics. This methodology can uncover concealed tendencies and guide focused initiatives to assist underprivileged populations ( Ponce et al., 2025; Victora et al., 2019). Finally, a sustainability gap remains, as little research addresses how policy, governance, and market linkages sustain CBL outcomes at scale.

A multidisciplinary perspective deepens these critiques. From the lens of education sciences, research emphasizes the role of pedagogy and scaffolding but rarely situates them in broader social and economic structures. Scaffolding is an extensively studied educational strategy that offers provisional assistance to students. It is based on constructivist theories and seeks to improve learning by rendering thought visible and facilitating knowledge building ( Mathé & Christensen, 2024). Sociology and anthropology highlight how trust, norms, and cultural authority mediate learning processes, yet these insights are seldom operationalized in program evaluation. In an Australian university, trust is established through dialogue and familiarity between academics and students, hence enhancing the reliability of assessments and facilitating learning ( McLean, 2018). Economics and development studies foreground employability and cost-effectiveness, while public health research links CBL to health literacy and collective action. Meanwhile, ICT4D scholarship draws attention to both the promise and pitfalls of digital mediation in extending access to CBL. Finally, governance studies underscore the critical importance of enabling policies and institutional frameworks in scaling community-based initiatives.

Taken together, these insights suggest that CBL should be understood not merely as a pedagogical method but as a systems intervention, requiring the co-evolution of pedagogy, social capital, market linkages, technology, and governance. Yet, the current literature tends to privilege single dimensions, producing fragmented insights and outcomes that are often short-lived.

## Methodology

### Research design and protocol

This study employed a Systematic Literature Review (SLR) design, guided by the Preferred Reporting Items for Systematic Reviews and Meta-Analyses (PRISMA 2020) framework. Adhering to PRISMA 2020 enhances the transparency of systematic reviews and meta-analyses, facilitating readers’ evaluation of the credibility and relevance of the results ( Page et al., 2021). The review protocol was established prior to the commencement of data collection and specified the research questions, eligibility criteria, search strategy, screening procedures, and synthesis methods. To enhance transparency and replicability, the study followed open-science best practices, and all related materials were deposited in Zenodo. This is essential in disciplines such as psychology and education, where compliance with standards like PRISMA and FAIR is stressed to improve reproducibility and data reutilization ( Burgard et al., 2025).

### Eligibility criteria

Eligibility criteria are established benchmarks utilized to ascertain which papers qualify for inclusion in a systematic review. These criteria are crucial for guaranteeing that the review is thorough and impartial ( Muñoz-Urtubia et al., 2023; Nagendrababu et al., 2023). The eligibility criteria were defined to ensure that only studies directly relevant to the effectiveness of community-based learning (CBL) were included. Studies were considered eligible if they were published in peer-reviewed journals, book chapters, or conference proceedings between 2010 and 2025, explicitly addressed CBL, learning communities, or community education, and reported measurable outcomes related to personal, social, or vocational skills. Empirical research employing quantitative, qualitative, or mixed-methods designs, as well as systematic or scoping reviews with explicit methodologies, was included.

Conversely, studies were excluded if they were non-empirical in nature, such as opinion pieces, commentaries, or editorials. Publications that lacked measurable outcomes within the three domains of interest were also excluded, as were studies limited to formal schooling contexts without a demonstrable community-based component. These criteria ensured that the final body of literature represented methodologically robust and contextually relevant evidence for assessing CBL effectiveness.

### Information sources and search strategy

A comprehensive search was conducted across five major academic databases Scopus, Pubmed, Crossref, Semantic Scholar, and Google Scholar selected for their broad coverage of education, social sciences, and development studies. Boolean operators and keyword combinations were applied to maximize retrieval. A representative search string was formulated as follows:

A core search strategy was developed and adapted to the indexing logic of each database using database-specific Boolean operators and controlled vocabulary where applicable. A representative search string is shown below, while full database-specific search strategies are provided in the supplementary materials (Zenodo repository).

(“community-based learning” OR “learning community” OR “community education”)

AND (“personal skills” OR “self-confidence” OR “self-efficacy”)

OR (“social skills” OR “teamwork” OR “civic engagement”)

OR (“vocational skills” OR “entrepreneurship” OR “technical training” OR “financial literacy”).

The search was restricted to English-language, peer-reviewed publications published between 2010 and 2025. Additionally, the reference lists of included articles were manually screened to identify further relevant studies not captured in the initial search.

### Selection process

The selection process was conducted in two stages. In the first stage, titles and abstracts were screened to remove duplicates and exclude irrelevant studies. In the second stage, full texts were reviewed to assess compliance with the eligibility criteria. It guarantees that the review is thorough, transparent, and methodologically rigorous, hence enhancing the quality of evidence synthesis ( Davis et al., 2021; Knobloch et al., 2011).

### Data extraction and quality appraisal

For each eligible study, data were systematically extracted on author, year of publication, country, research context, study design, outcomes assessed, and indicators used to measure effectiveness. The methodological quality of included studies was evaluated using the Joanna Briggs Institute (JBI) Critical Appraisal Checklists and the Critical Appraisal Skills Programme (CASP) tools, tailored to study design. Employing both JBI and CASP instruments guarantees a thorough assessment of methodological quality. The JBI tools are very beneficial for qualitative research and systematic reviews, whilst CASP tools are proficient for the swift appraisal of Level I evidence, including systematic reviews and meta-analyses ( Chang et al., 2022; Salins et al., 2024). This dual approach ensured both internal validity and consistency across diverse methodologies.

### Synthesis

The extracted data were analyzed using thematic synthesis, enabling the organization of findings into categories such as indicators of effectiveness, personal outcomes, social outcomes, vocational outcomes, and contextual factors. A narrative synthesis approach was adopted to compare patterns and divergences across studies, while highlighting consistencies and contradictions in the evidence base. Narrative synthesis is an effective method for integrating varied and intricate material, providing comprehensive contextual insights and an enhanced comprehension of research outcomes. Nonetheless, it necessitates meticulous implementation of structured frameworks to guarantee transparency and rigor ( Madden et al., 2018, 2019).

### Ethical considerations

As this research was based solely on secondary data from published sources, no primary data collection was undertaken, and therefore, ethical approval was not required. Nevertheless, the review adhered to established guidelines for transparency, rigor, and research integrity.

## Results

### Study selection

**Figure 1.**
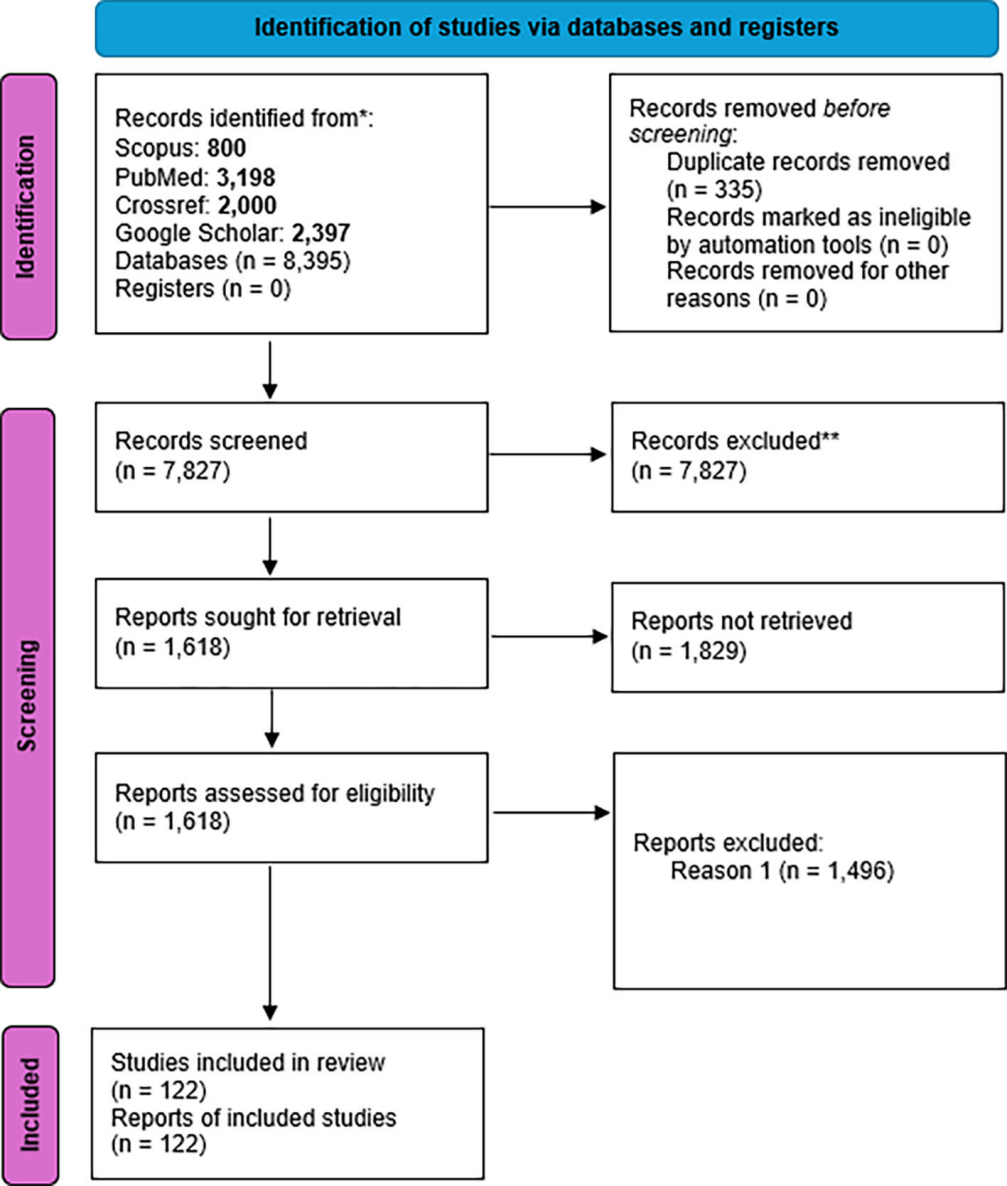
PRISMA 2020 flow diagram—Community-based learning: personal, social, and vocational outcomes (2010–2025).

The PRISMA 2020 flow diagram ( Figure 1) summarizes the selection process. Across four databases (Scopus, PubMed, Crossref, and Google Scholar), we identified 8,395 records. After removing 335 duplicates, 8,060 unique records remained for title-and-abstract screening. We sought retrieval for 3,447 reports and successfully retrieved 1,618 full texts (1,829 were not retrieved). Following full-text assessment, 1,496 reports were excluded (predominantly for lacking an explicit community-based component), leaving 122 studies for inclusion in the synthesis (see Figure 1).

### Characteristics of included studies

The 122 included studies span multiple regions and CBL modalities (e.g., community learning centres/PKBM, women’s cooperatives, peer-mentoring networks, service-learning, place-based STEM). Designs range from qualitative case studies and mixed-methods evaluations to quasi-experimental and longitudinal cohorts. Outcomes cluster around personal (e.g., self-efficacy, agency, self-regulation), social (e.g., teamwork, leadership, civic engagement, trust), and vocational domains (e.g., employability, entrepreneurship, financial literacy). Table 2 summarizes study descriptors.

**Table 2.** Summarizes the characteristics of the included studies.

Author(s), Year	Country/Region	Study design	CBL context	Outcomes measured	Domains (P/S/V) *
Zamiri & Esmaeili, 2024	Iran	Mixed-methods	Peer mentoring & collaborative projects in community settings	Confidence, teamwork, social cohesion	P, S
Harmsel-Nieuwenhuis et al., 2025	Netherlands	Longitudinal evaluation	Community-based life-skills for vulnerable youth	Transfer of life skills to vocational capacities	P, V
Kizys et al., 2025	UK (rural STEM)	Quasi-experimental	Place-based CBL for rural STEM learners	Career readiness, social engagement	S, V
Raniga, 2021	South Africa	Qualitative case study	Women’s sewing cooperatives	Income, solidarity, empowerment	S, V
Wiyono et al., 2021	Indonesia	Survey + case	Community Learning Centers (PKBM/CLC)	Access to education, vocational training	V
Lake, 2021	USA	Longitudinal cohort	Service-learning in higher education	Civic engagement, professional skills	P, S
Arthur-Holmes, 2024	Ghana (ASM sector)	Ethnography	Informal women’s cooperatives	Collective action, empowerment	S
Belete, 2022	Asia (multi-country ALE/CLCs)	Review/empirical synthesis	Adult Learning & Education via CLCs	Literacy, technical/vocational skills	V
Bun et al., 2025	Cambodia	Case/mixed evaluation	CLCs during COVID-19	Continuity of vocational education	V
UN Women, 2023	Ethiopia	Program evaluation/report	Women’s cooperative with life-skills & finance	Livelihoods, basic business/life skills	V, S

* P = Personal, S = Social, V = Vocational.

### Thematic findings

**Indicators of effectiveness**

Operationalizations of CBL effectiveness commonly included participation/engagement, collaboration/communication, and contextual relevance, alongside learner-centred constructs (e.g., self-efficacy, motivation) and labour-market-proximal outcomes (technical competence, entrepreneurial activity, employability). This diversity demonstrates CBL’s versatility but complicates cross-study comparability, motivating a unified indicator set and standardized reporting.

**Personal outcomes**

Across youth and adult populations, CBL is consistently associated with gains in self-confidence, agency, self-regulation, discipline, and responsibility. Authentic, community-situated tasks appear to foster stronger ownership and resilience than classroom-only comparators. Where longitudinal data exist, improvements tend to persist, though durability beyond program cycles remains under-tested.

**Social outcomes**

The participatory nature of CBL strengthens teamwork, leadership, trust, civic engagement, and social cohesion, frequently via Communities-of-Practice dynamics (participation → identity → social capital). Students gain leadership and project management skills, as well as cooperative and conflict resolution skills, from this cooperative method ( DelSesto et al., 2025; Stevahn et al., 2016). Several contexts show spillovers from individual growth to collective efficacy and solidarity, particularly in cooperatives and community learning centres.

**Vocational outcomes**

Evidence for employability, entrepreneurship, and financial capabilities is positive but context dependent. Effects are strongest where programs embed market linkages, mentoring, and work based projects; conversely, short cycles and weak employer engagement attenuate impacts. Few studies track long-term placement, income, or enterprise survival, limiting conclusions about durability. Studies on long-term foster care placements reveal favorable results in familial and social interactions, as well as pro-social behaviors throughout time, notwithstanding apprehensions over placement stability and academic performance ( Fernandez, 2009).

**Supporting and hindering factors**

Effectiveness is enabled by co-design with communities, facilitator competence, cultural fit, partnerships (industry/NGO), supportive policy, and adequate infrastructure (including digital). Barriers include fragmented indicators, short-term funding, uneven governance/monitoring, digital divides, and gaps in equity reporting. Overall, CBL functions as a **s**ystem intervention whose success depends on pedagogy and the surrounding ecosystem. This encompasses active learning methodologies, competency-based evaluations, educator training, technological incorporation, and institutional backing. Tackling these components can facilitate the resolution of difficulties and guarantee that CBL adequately equips students for practical applications ( Fernandez, 2009; Ford, 2024).

**Synthesis**

Using a structured narrative approach, we grouped outcomes by personal, social, and vocational domains and standardized direction-of-effect (benefit /no clear effect/harm). Patterns favour CBL for personal and social outcomes, with positive but more variable signals for vocational outcomes. We interpret these patterns cautiously, noting heterogeneity in measures and designs and the constraints of direction-based synthesis. An equity lens suggests disproportionate benefits for women, rural learners, and low-SES groups in programs with inclusive design and economic linkages.

**Mapping of conceptual and evaluative models**

The synthesis indicates that although explicit evaluative frameworks are rarely articulated in the reviewed literature, three dominant conceptual logics can be identified across studies.

First, capability-oriented approaches conceptualize community-based learning as the expansion of individual agency and freedoms. These studies emphasize outcomes such as self-efficacy, autonomy, and empowerment as indicators of enhanced capability.

Second, participation-based models, aligned with communities-of-practice and dialogical learning perspectives, frame empowerment as identity formation and collective engagement. Here, evaluative emphasis is placed on collaboration, leadership, trust, social capital, and civic participation.

Third, vocational transition models link community-based learning to labor-market integration and economic sustainability. These approaches evaluate effectiveness through employability, entrepreneurial activity, technical competence, and income-related indicators.

While few studies explicitly integrate these strands into unified evaluative models, the synthesis suggests that effective CBL initiatives operate at the intersection of capability expansion, participatory identity formation, and vocational transition mechanisms.

## Discussion

### Interpretation of research questions

The synthesis indicates that Community-Based Learning (CBL) reliably improves personal and social outcomes, while vocational outcomes are positive yet more contingent on context. Authentic, community situated tasks appear to cultivate self confidence, agency, self regulation, and a stronger sense of responsibility capacities that sustain learning beyond classrooms. Socially, participation and collaboration foster teamwork, leadership, trust, cohesion, and civic engagement; individual gains frequently reverberate into collective efficacy. Team collaboration enables members to exchange skills, talents, and ideas, resulting in enhanced efficiency, innovation, and strengthened interpersonal ties ( Geada, 2023; Weil et al., 2017). Vocational effects employability, technical competence, financial capability, and entrepreneurial orientation strengthen when programs are anchored to market pathways (mentoring, internships, enterprise incubation) and weaken under short cycles or minimal employer engagement. Across studies, indicators used to assess effectiveness are diverse and unevenly specified, underscoring the need for a core indicator set that enables comparability.

### Key success factors

CBL succeeds when several conditions co-occur. Co-design with communities secures relevance and ownership. Co-design guarantees that initiatives are directly pertinent to the community’s requirements, hence enhancing their efficacy and sustainability ( Lam et al., 2017; Murdock et al., 2023). Facilitator capability especially dialogic pedagogy, individualized coaching, and boundary spanning between education providers, communities, and employers acts as a principal lever. Cultural and linguistic fit builds trust; stable governance and financing prevent program drift; and cross-sector partnerships (including industry and NGOs) translate learning into opportunities. Digital mediation can widen access and scaffold reflection, provided inclusion safeguards address connectivity and device gaps. Above all, disciplined measurement minimum outcomes per domain and routine equity disaggregation enables programs to learn and improve.

### Theoretical integration

Findings support a capability in practice account of CBL. Dialogue and participation enlarge agency; expanding agency, in turn, enables deeper participation creating a reinforcing loop. The interaction between dialogue and agency is apparent in organizational contexts when genuine leadership cultivates a dialogue-centric culture, hence enhancing learning and agency across various levels within the organization ( Mazutis & Slawinski, 2008). As participants enter shared practices, identities and social capital form, which opens vocational opportunities. Adult-learning conditions (autonomy, relevance, application) furnish the pedagogical substrate across age groups. CBL therefore functions less as a classroom method and more as a systems intervention: pedagogical mechanisms (participation, collaboration, relevance) operate through institutional and structural mediators (culture, facilitator quality, digital infrastructure, governance, market linkages) to generate mutually reinforcing personal, social, and vocational outcomes that endure when sustainment provisions are in place.

### Novelty of the study

This review contributes by (i) integrating personal, social, and vocational outcomes within a single analytic lens; (ii) proposing an indicator–instrument map that improves cross-study comparability; (iii) applying an explicit equity lens to examine who benefits most; and (iv) operationalizing a transparent, appraisal-aware synthesis approach that remains informative amid heterogeneity.

### Practical implications

Programs should begin with aset based co-design and task authenticity that ties competencies to real problems. Facilitator development must be planned upfront covering dialogic methods, mentoring, project design, and partnership orchestration. Facilitators must be processually adaptive, balancing support and challenge to sustain a dialogic environment marked by inquiry and exploration ( Alrø & Billund, 2021). To maximize vocational effects, learning should be coupled with market pathways (structured internships, micro-enterprise support, employer co-mentoring). Institutionalization through community learning centers or comparable structures requires financing envelopes, light touch accreditation, and data systems capable of tracking medium-term outcomes. Finally, equity by design should be embedded: flexible schedules, transport/childcare supports, accessibility features, and safe participation channels for underserved groups.

### Theoretical implications

The results clarify why vocational outcomes often materialize after gains in personal and social domains: employability and entrepreneurship are more likely when agency, practice based identity, and social capital have first taken root. The proposed framework enables multilevel theory testing micro (facilitator–learner interactions), meso (community networks and institutions), and macro (policy, markets, digital infrastructures) and yields testable propositions (e.g., participation → agency → social capital → employability), moderated by market linkages and governance quality.

### Limitations

Heterogeneity of designs, populations, and measures constrained meta-analysis and necessitated direction of effect synthesis. Indicator variability and inconsistent equity reporting reduce comparability and risk obscuring distributional effects. Many studies adopt short time horizons; evidence on durability job placement, income stability, or enterprise survival remains limited. A separate study examined the influence of supervised work placements on career outcomes, concluding that these placements typically yield a favorable impact on employment, facilitating quicker job acquisition for graduates and improving their self-efficacy, knowledge, abilities, and attitudes ( Inceoglu et al., 2019). Language scope and time window may omit relevant local evidence. In several instances, eligibility decisions depended on available links or metadata, which can undercount community components not visible in titles or abstracts.

### Directions for future research

Future work should (1) standardize outcomes via a core indicator set for each domain with validated instruments and mandatory equity disaggregation; (2) strengthen causal inference through longitudinal and quasi experimental designs, realist evaluation, and process tracing; (3) track durability over 12–36 months for placement, income, and enterprise outcomes; (4) interrogate digital mediation to identify blends that expand access without deepening divides; (5) model ecosystem interactions among governance, financing, and market linkages, including cost effectiveness; (6) compare modalities (service-learning, community learning centers, cooperative-based, place-based STEM) to identify threshold conditions in low-resource and rural settings; and (7) improve reporting quality (registered protocols, shareable extraction sheets, subgroup reporting) to enable higher-precision quantitative synthesis.

## Conclusion

### Summary

This review shows that Community-Based Learning (CBL) functions as a multidomain empowerment strategy. Evidence converges on consistently positive effects for personal and social outcomes such as self-confidence, agency, self-regulation, teamwork, leadership, trust, and civic engagement while vocational impacts are positive yet more contingent on context. Vocational gains strengthen when programs embed market linkages, mentoring, and authentic work-based projects; they weaken under short cycles or minimal employer engagement. Heterogeneity in study designs and outcome measures limited meta-analysis and accentuated the need for standardized indicators and equity-aware reporting. Taken together, the findings support reading CBL not merely as a pedagogical technique but as a systems intervention whose effectiveness depends on the alignment of pedagogy with culture, governance, digital infrastructure, and labor-market connections. The cultural fit of CBL and the backing of governing bodies are essential to its success. For example, in order to improve student abilities, the application of CBL in nursing education in Cambodia necessitated cultural adaptation and assistance from educational institutions and clinical preceptors ( Koto-Shimada et al., 2023).

### Contributions

This study advances the field in four ways. First, it integrates personal, social, and vocational outcomes within a single analytic lens, countering the fragmentation that dominates prior work. Second, it proposes a unified indikator instrument map that clarifies what to measure and how, thereby improving comparability across settings. Third, it brings an explicit equity lens to bear on CBL, highlighting who benefits most and where reporting gaps persist.

Fourth, it demonstrates a transparent, appraisal-aware synthesis approach and systematically categorizes dominant conceptual and evaluative logics underlying CBL implementation, thereby fulfilling the stated objective of model mapping.

### Recommendations

To translate these insights into action, programs should co-design with communities, ensure facilitator capability in dialogic and partnership oriented practice, and hard-wire market pathways (structured internships, employer co-mentoring, micro-enterprise incubation) into the learning architecture. Institutions and policymakers should institutionalize CBL through stable financing, light touch accreditation, and interoperable data systems capable of tracking medium-term outcomes and equity subgroups. Researchers and evaluators should adopt a core indicator set for personal, social, and vocational domains, use validated instruments, register protocols, and prioritize longitudinal and quasi-experimental designs that test mechanisms and durability over 12–36 months. By aligning pedagogy with ecosystem supports and by standardizing how outcomes are measured and reported, the field can move from promising projects to scalable, equitable, and sustained community-based learning systems. Community-based learning is acknowledged as an effective pedagogical approach that promotes the common good and improves educational environments by merging classroom instruction with community service and action ( Nesmith et al., 2020; Zlotkowski & Duffy, 2010).

## Use of Generative AI

During the preparation and revision of this manuscript, the authors used ChatGPT (OpenAI, GPT-5.2 version) to assist with language refinement, structural clarity, and editorial improvement of selected sections. The tool was used solely to enhance readability and organization of the manuscript in response to peer-review feedback. All conceptual development, methodological design, data analysis, interpretation of findings, and final content decisions were conducted by the authors. The authors take full responsibility for the accuracy, integrity, and originality of the work.

## PRISMA Checklist & Flowchart

Zenodo. Beyond Classrooms: Community-Based Learning for Personal, Social, and Vocational Empowerment. ( https://zenodo.org/records/17499617) Ridwan, I. et al. (2025).

This project contains the following underlying data:

• PRISMA_Checklist.xlsx: Completed PRISMA checklist
• PRISMA_Flowchart.tif: Flowchart illustrating the screening and selection of studies

Data is available under the terms of the CC BY 4 license.

## Data Availability

Zenodo. Beyond Classrooms: Community-Based Learning for Personal, Social, and Vocational Empowerment. ( https://zenodo.org/records/17499617) Ridwan, I. et al. (2025). This project contains the following underlying data:
• PRISMA_Checklist.xlsx – Completed PRISMA 2020 checklist used for this systematic review. • PRISMA_Flowchart.tif – PRISMA 2020 flow diagram illustrating search, screening, and inclusion of studies. •
Extracted_Study_Data.xlsx – Data extraction sheet containing study descriptors, outcomes, and indicators used in the synthesis. • Graph_Data.xlsx – Dataset containing values used to generate figures and direction-of-effect visuals. •
Image_Extraction_Data.xlsx – Points extracted from study figures for cross-visual analysis. Data is available under the terms of the CC BY 4 license.
