# Supplementary material for: Beyond Classrooms: Community-Based Learning for Personal, Social, and Vocational Empowerment
Source: F1000Res. 2026 Jun 9;14:1274. Originally published 2025 Nov 18. [Version 3] doi: 10.12688/f1000research.171496.3 (PMC13250508; doi:10.12688/f1000research.171496.3)
Supplement: Peer review history

# Reviewer report: Reviewer response for version 2

**DOI:** 10.5256/f1000research.196515.r488887

**Published:** 2026-06-09

**Authors:** Febblina Daryanes

**Affiliations:** 1 Universitas Riau (Ringgold ID: 175475), Pekanbaru, Riau, Indonesia

I have reviewed the revised version of the manuscript and the author's responses to the reviewers' comments. The author has provided adequate responses and made necessary improvements based on the feedback provided in the previous round of review.

Are the rationale for, and objectives of, the Systematic Review clearly stated?

Yes

Is the statistical analysis and its interpretation appropriate?

Yes

If this is a Living Systematic Review, is the ‘living’ method appropriate and is the search schedule clearly defined and justified? (‘Living Systematic Review’ or a variation of this term should be included in the title.)

Not applicable

Are sufficient details of the methods and analysis provided to allow replication by others?

Yes

Are the conclusions drawn adequately supported by the results presented in the review?

Partly

Reviewer Expertise:

Education

I confirm that I have read this submission and believe that I have an appropriate level of expertise to confirm that it is of an acceptable scientific standard.

# Reviewer report: Reviewer response for version 1

**DOI:** 10.5256/f1000research.189112.r482316

**Published:** 2026-05-20

**Authors:** Sriyanti Mustafa

**Affiliations:** 1 Universitas Muhammadiyah Parepare, South Sulawesi, Indonesia

This article provides a valuable and timely systematic review of Community-Based Learning (CBL) and its contributions to personal, social, and vocational empowerment. The manuscript is well-structured, theoretically grounded, and supported by a broad range of international literature. The integration of personal, social, and vocational outcomes into a single analytical framework represents an important contribution to the field of community-based and lifelong learning.

 The methodology generally follows PRISMA 2020 guidelines, and the review process is adequately described. The findings are clearly synthesized and demonstrate consistent positive impacts of CBL across multiple educational and community contexts. The discussion effectively connects the findings with educational practice, community empowerment, and policy implications.

 The manuscript would benefit from several minor improvements, particularly clearer harmonization of database descriptions between sections, more detailed database-specific search strategies, and a more explicit presentation of conceptual or evaluative frameworks discussed in the review. These suggestions are intended to strengthen transparency and readability rather than address major methodological concerns.

 Overall, this study makes a meaningful contribution to the literature on community-based learning, empowerment, and lifelong education and is suitable for indexing.

Are the rationale for, and objectives of, the Systematic Review clearly stated?

Yes

Is the statistical analysis and its interpretation appropriate?

Yes

If this is a Living Systematic Review, is the ‘living’ method appropriate and is the search schedule clearly defined and justified? (‘Living Systematic Review’ or a variation of this term should be included in the title.)

Not applicable

Are sufficient details of the methods and analysis provided to allow replication by others?

Yes

Are the conclusions drawn adequately supported by the results presented in the review?

Yes

Reviewer Expertise:

Community-Based Learning; Educational Research; Inclusive Pedagogy; Lifelong Learning

I confirm that I have read this submission and believe that I have an appropriate level of expertise to confirm that it is of an acceptable scientific standard.

# Response

**Published:** 2026-05-22

**Authors:** Ihwan Ridwan

**Affiliations:** Universitas Negeri Makassar, Makassar, South Sulawesi, Indonesia

Thank you very much for the constructive and encouraging review. We sincerely appreciate the positive evaluation of our manuscript and the valuable suggestions provided. In response to the reviewer’s comments, we have carefully revised the manuscript by improving the consistency of database descriptions across sections, clarifying database-specific search strategies to enhance transparency and replicability, and strengthening the explicit presentation of the conceptual and evaluative framework discussed in the review. We believe these revisions have improved the clarity, coherence, and overall quality of the manuscript.

# Response

**Published:** 2026-06-01

**Authors:** Ihwan Ridwan

**Affiliations:** Universitas Negeri Makassar, Makassar, South Sulawesi, Indonesia

We sincerely thank the reviewer for the positive and encouraging evaluation of our manuscript. We greatly appreciate the recognition of this study’s contribution to the literature on community-based learning, empowerment, and lifelong education, as well as the acknowledgment of its methodological rigor and overall suitability.

 We are pleased that the rationale, objectives, methods, analysis, and conclusions were considered clearly presented and adequately supported. In response to the constructive suggestions provided, we have implemented several minor refinements to further improve transparency and readability, particularly by harmonizing database descriptions across sections and clarifying methodological reporting to ensure stronger consistency throughout the manuscript.

 We deeply appreciate the reviewer’s thoughtful assessment and valuable support, which has helped strengthen the clarity and presentation of this work.

 Thank you again for your time, expertise, and constructive feedback.

# Reviewer report: Reviewer response for version 1

**DOI:** 10.5256/f1000research.189112.r454056

**Published:** 2026-02-11

**Authors:** Febblina Daryanes

**Affiliations:** 1 Universitas Riau (Ringgold ID: 175475), Pekanbaru, Riau, Indonesia

1. Are the rationale for, and objectives of, the Systematic Review clearly stated?

 Answer: Yes

 The rationale and objectives are clearly articulated and logically connected. The authors convincingly argue the need to synthesize evidence on community-based learning (CBL) across personal, social, and vocational domains, which are often examined separately. The objectives are explicitly stated and aligned with the identified gap in the literature.

 However, the framing of the rationale would benefit from engagement with a broader and more up-to-date body of international literature, particularly recent high-impact systematic reviews and conceptual works on experiential, community-engaged, and vocational learning, to further strengthen the scholarly positioning of the study.

 2. Are sufficient details of the methods and analysis provided to allow replication by others?

 Answer: Yes

 The review follows PRISMA 2020 guidelines and provides a general description of databases searched, inclusion/exclusion criteria, and screening procedures. The use of established appraisal tools (JBI and CASP) is appropriate.

 Nevertheless, full reproducibility is limited by:

 the absence of complete search strings for each database, inconsistencies between the databases listed in the abstract and those described in the methods, and limited detail regarding duplicate removal and full-text retrieval procedures.

 Providing full search strategies and harmonizing database descriptions would substantially improve replicability.

 The rationale for limiting the search to studies published from 2010 onwards is not clearly explained. As temporal restrictions in systematic reviews can influence the scope and interpretation of findings

 3. Is the statistical analysis and its interpretation appropriate?

 Answer: yes

 While the authors report the use of established quality appraisal tools (JBI and CASP), the methods section would benefit from a clearer explanation of how potential biases were minimized during data extraction and synthesis. In particular, it is not explicitly stated whether data extraction and thematic synthesis were conducted independently by multiple reviewers, how disagreements were resolved, or how study quality informed the interpretation of findings. Providing greater transparency on these procedures would strengthen confidence in the robustness and trustworthiness of the narrative synthesis.

 4. Are the conclusions drawn adequately supported by the results presented in the review?

 Answer: partly

 The conclusions are generally supported by the synthesized findings presented. However, given that some stated objectives particularly the systematic mapping of indicators and conceptual or evaluative models were only partially addressed, the conclusions could be further strengthened by more explicitly linking them to these unmet aims.

 Overall, the article addresses objectives (i) and (iii) satisfactorily, partially addresses objective (ii), and does not fully achieve objective (iv). In particular, while the review discusses conceptual themes related to community-based learning, it stops short of systematically mapping or synthesizing existing conceptual or evaluative models, as stated in the aims. Addressing this gap ideally through a visual conceptual framework or explicit model mapping would significantly strengthen alignment between the study’s objectives and its reported outcomes.

Are the rationale for, and objectives of, the Systematic Review clearly stated?

Yes

Is the statistical analysis and its interpretation appropriate?

Yes

If this is a Living Systematic Review, is the ‘living’ method appropriate and is the search schedule clearly defined and justified? (‘Living Systematic Review’ or a variation of this term should be included in the title.)

Not applicable

Are sufficient details of the methods and analysis provided to allow replication by others?

Yes

Are the conclusions drawn adequately supported by the results presented in the review?

Partly

Reviewer Expertise:

Education

I confirm that I have read this submission and believe that I have an appropriate level of expertise to confirm that it is of an acceptable scientific standard, however I have significant reservations, as outlined above.

# Response

**Published:** 2026-04-21

**Authors:** Ihwan Ridwan

**Affiliations:** Universitas Negeri Makassar, Makassar, South Sulawesi, Indonesia

Thank you very much for your thorough and constructive review. We sincerely appreciate the positive evaluation of the scientific quality of our manuscript and your valuable suggestions for improvement. We have carefully revised the manuscript and addressed all points raised as follows:

**1. Rationale and Objectives**

 We have strengthened the Introduction and Literature Review sections by incorporating more recent and high-impact international studies on community-based learning, experiential learning, and vocational education. These additions enhance the scholarly positioning of the study and provide a broader global context for our review.

**2. Replicability of Methods**

 We have improved methodological transparency by:

- Providing complete search strings for each database in the supplementary materials (Zenodo repository)
- Harmonizing the database descriptions across the Abstract, Methods, and Results sections
- Expanding the selection process to clearly describe duplicate removal and full-text screening procedures
- Adding a clear justification for the 2010–2025 time frame, reflecting the emergence of contemporary CBL practices aligned with SDG-oriented learning and vocational empowerment

**3. Bias Minimization and Analytical Rigor**

 The Methods section has been revised to explicitly clarify that:

- Data extraction and thematic synthesis were conducted independently by multiple reviewers
- Discrepancies were resolved through discussion and consensus
- Study quality appraisal (JBI and CASP) informed the interpretive weighting of findings

These revisions strengthen the transparency and robustness of the synthesis process.

**4. Alignment of Objectives and Conceptual Model Mapping**

 In response to this important comment, we have added a new subsection entitled **“Mapping of Conceptual and Evaluative Models”** in the Results section. This section systematically categorizes three dominant conceptual logics underlying CBL:

- Capability-oriented models
- Participation-based models (communities of practice/dialogical learning)
- Vocational transition models

This addition directly addresses objective (iv) and provides a structured model-mapping synthesis rather than a descriptive discussion. The Conclusion and Contributions sections have also been revised to explicitly reflect this alignment.

 Overall, we believe that these revisions substantially improve the clarity, methodological rigor, and theoretical contribution of the manuscript.

 Thank you again for your insightful feedback, which has significantly strengthened this work.

# Response

**Published:** 2026-06-01

**Authors:** Ihwan Ridwan

**Affiliations:** Universitas Negeri Makassar, Makassar, South Sulawesi, Indonesia

We sincerely thank the reviewer for the thoughtful, constructive, and detailed evaluation of our manuscript. We greatly appreciate the recognition of the study’s rationale, theoretical grounding, and overall contribution to the literature on Community-Based Learning (CBL). In response to the valuable suggestions provided, we have carefully revised the manuscript to further improve transparency, consistency, and alignment between sections.

**1. Rationale and Objectives**

 We appreciate the reviewer’s positive assessment of the clarity and coherence of the study rationale and objectives. To further strengthen the scholarly positioning of the review, we refined the framing of the manuscript through clearer alignment between the review objectives and the conceptual contributions synthesized in the study.

**2. Replicability of Methods and Analysis**

 In response to the reviewer’s recommendations regarding reproducibility and transparency, we revised the Methodology section to improve clarity and consistency. Specifically, database descriptions have been harmonized across sections to ensure internal consistency, and the search strategy description has been strengthened by clarifying the use of database-adapted search approaches. We also explicitly indicate that full database-specific search strategies are available in the supplementary materials (Zenodo repository), thereby improving transparency and reproducibility.

**3. Analytical Transparency and Trustworthiness**

 Following the reviewer’s recommendation, we refined the methodological explanation to provide clearer reporting of the synthesis process and strengthen interpretive transparency in the review.

**4. Alignment Between Objectives and Conclusions**

 We appreciate the reviewer’s important observation regarding the presentation of conceptual and evaluative frameworks. To improve alignment with the stated objectives, the manuscript now presents a clearer and more explicit articulation of the conceptual and evaluative dimensions synthesized across studies, including stronger integration between the Results, Abstract, and Conclusion sections.

 Overall, these revisions were undertaken to improve clarity, consistency, methodological transparency, and alignment between the study objectives and reported findings. We sincerely appreciate the reviewer’s constructive feedback, which has helped strengthen the manuscript.

 Thank you again for your time, expertise, and valuable comments.
